# Neurocardiovascular deficits in the Q175 mouse model of Huntington's disease

**DOI:** 10.14814/phy2.13289

**Published:** 2017-06-02

**Authors:** Tamara S. Cutler, Saemi Park, Dawn H. Loh, Maria C. Jordan, Tomohiro Yokota, Kenneth P. Roos, Cristina A. Ghiani, Christopher S. Colwell

**Affiliations:** ^1^ Department of Psychiatry & Biobehavioral SciencesUniversity of California, Los AngelesLos AngelesCalifornia; ^2^ Department of Physiology and Cardiovascular Research LabUniversity of California, Los AngelesLos AngelesCalifornia; ^3^ Department of AnesthesiologyDivision of Molecular MedicineDavid Geffen School of MedicineUniversity of California, Los AngelesLos AngelesCalifornia; ^4^ Department of Pathology & Laboratory MedicineUniversity of California, Los AngelesLos AngelesCalifornia

**Keywords:** Autonomic nervous system, baroreceptor reflex, cardiovascular function, circadian rhythms, core body temperature, echocardiograms, electrocardiograms, fibrosis, heart rate variability, Huntington's disease, radio telemetry

## Abstract

Cardiovascular dysautonomia as well as the deterioration of circadian rhythms are among the earliest detectable pathophysiological changes in individuals with Huntington's disease (HD). Preclinical research requires mouse models that recapitulate disease symptoms and the Q175 knock‐in model offers a number of advantages but potential autonomic dysfunction has not been explored. In this study, we sought to test the dual hypotheses that cardiovascular dysautonomia can be detected early in disease progression in the Q175 model and that this dysfunction varies with the daily cycle. Using radiotelemetry implants, we observed a significant reduction in the diurnal and circadian activity rhythms in the Q175 mutants at the youngest ages. By middle age, the autonomically driven rhythms in core body temperature were highly compromised, and the Q175 mutants exhibited striking episodes of hypothermia that increased in frequency with mutant *huntingtin* gene dosage. In addition, Q175 mutants showed higher resting heart rate (HR) during sleep and greatly reduced correlation between activity and HR. HR variability was reduced in the mutants in both time and frequency domains, providing more evidence of autonomic dysfunction. Measurement of the baroreceptor reflex revealed that the Q175 mutant could not appropriately increase HR in response to a pharmacologically induced decrease in blood pressure. Echocardiograms showed reduced ventricular mass and ejection fraction in mutant hearts. Finally, cardiac histopathology revealed localized points of fibrosis resembling those caused by myocardial infarction. Thus, the Q175 mouse model of HD exhibits cardiovascular dysautonomia similar to that seen in HD patients with prominent sympathetic dysfunction during the resting phase of the activity rhythm.

## Introduction

Huntington's disease (HD) patients suffer from a progressive neurodegenerative process that inflicts cognitive, psychiatric and motor dysfunction (Margolis and Ross 2003; Kuljis et al. 2012a). HD is caused by a CAG repeat expansion within the first exon of the *Huntington* (*Htt*) gene which produces a polyglutamine repeat that leads to protein misfolding, soluble aggregates, and inclusion bodies detected throughout the body (Saft et al. 2005; Ciammola et al. 2006). The normal function of the protein (HTT) is unknown; however, the mutated form leads to dysfunction of a large range of cellular processes including cytoskeletal organization, protein folding, metabolism and transcriptional activities. Cardiovascular events are a major cause of early death in the HD population and occur at a higher rate compared to the rest of the population (Chiu and Alexander 1982; Lanska et al. 1988; Sørensen and Fenger 1992). Individuals with HD commonly exhibit autonomic nervous system (ANS) dysfunction that can be detected early in the disease progression (Sharma et al. 1999; Andrich et al. 2002; Kobal et al. 2004; Bär et al. 2008; Aziz et al. 2010) and may be responsible for the high incidence of cardiovascular disease and early mortality due to cardiovascular events in the HD population.

Mouse models of HD recapitulate aspects of the human disease (Pouladi et al. 2013) including cardiovascular dysfunction. For example, ANS dysfunction has been reported in HD models as measured by heart rate variability (HRV) and baroreceptor reflex experiments (Kudo et al. 2011; Schroeder et al. 2011; Kiriazis et al. 2012; Mielcarek et al. 2014). Reduced contractility and cardiac output are also common features in HD mouse models (Mihm et al. 2007a; Kudo et al. 2011; Schroeder et al. 2011; Wood et al. 2012; Buonincontri et al. 2014). The cardiac‐specific expression of polyQ repeats leads to cardiovascular dysfunction, suggesting that cardiovascular disease may be the result of cardiomyocyte abnormalities as well as improper ANS input (Pattison et al. 2008; Melkani et al. 2013). One of the noteworthy aspects of the cardiovascular malfunction uncovered in previous work is that there is a time of day influence with hypertension emerging during the normal sleep time (Schroeder et al. 2011, 2016). This intersection between cardiovascular symptoms and the circadian cycle needs to be better explored in preclinical models if we are to develop a mechanistic understanding of the neurocardiovascular pathology.

Among the mouse models of HD, the heterozygote (Het) Q175 offers advantages including: a single copy of the mutation, genetic precision of the insertion and control of mutation copy number (Bode et al. 2009; Oakeshott et al. 2011). These features make the Het Q175 line perhaps the most clinically relevant among the animal models; however, possible autonomic dysfunction has not been examined. In this study, we sought to test the dual hypotheses that cardiovascular dysautonomia can be detected early in disease progression in the Q175 model and that this dysfunction varies with the daily cycle. We used radiotelemetry to assess diurnal and circadian rhythms in activity, core body temperature (CBT), and heart rate (HR) in young (3–4 months of age) and middle aged (9–10 months of age) Q175 and wild‐type (WT) mice. To further explore possible autonomic dysfunction, we measured HRV throughout the 24‐h cycle as well as the baroreceptor reflex during the normal rest cycle. In addition, we evaluated the age‐dependent progression in heart dysfunction in a separate cohort of mice using echocardiograms starting at 3 months of age and progressing to 12 months of age when the motor symptoms are pronounced and brain atrophy can be detected. Tissue was then collected to examine histoanatomical features of the Q175 hearts.

## Materials and Methods

### Ethical approval

Homozygous (Hom) and Het Q175 mice on the C57BL6/J background along with littermate WT controls were acquired from the mouse‐mutant resource at The Jackson Laboratory, (JAX, Bar Harbor, Maine; stock No: 370476). The mice are full backcrossed into the C57 line (>12 crosses) and are monitored for the number of CAG repeats (approximately 190). Het Q175 mice commonly live to 24 months of age but the health of the Hom Q175 declines precipitously after 16 months. Therefore, we stopped this study well before this age point. Three separate cohorts of mice of each genotype were used for this study: (1) Telemetry studies measuring activity, CBT and electrocardiograms (ECG). Recordings (6–8 weeks in duration) were made from mice in a light‐dark (LD) cycle as well as in constant darkness (DD) starting when the mice were 3 months and then again at 9 months of age; (2) Baroreceptor reflex measurements at 9 months of age; (3) longitudinal measurement of cardiac function using echocardiograms with measurements made at 3, 6, 9 and 12 months. The hearts of this third cohort of mice were then used for histological analysis. The LD cycle consisted of 12 h of light and 12 h of dark. By convention, the time of lights‐on being defined as zeitgeber time zero (ZT 0). By convention, when the mice were held in DD, the beginning of activity onset is defined at circadian time zero (CT 0).

The health of the mice was monitored daily. We looked for a set of symptoms including restlessness, impaired mobility, licking or wound guarding, failure to groom, open sores, loss of appetite or weight loss. At any sign of ill health, a member of the veterinary staff was consulted for course of treatment. If the animal did not improve, then it would be humanely sacrificed with an overdose of isoflurane followed by decapitation. For postsurgical pain management (telemetry cohort), the mice were given carprofen, 5 mg/kg every 24 h for 48 h minimum. The first dose was given before surgery, and the remaining doses postoperatively. All procedures followed guidelines of the National Institutes of Health and were approved by the UCLA Animal Research Committee.

### Telemetry measurements

Methods employed were similar to those previously described (Kudo et al. 2011; Schroeder et al. 2016). WT, Hom Q175, and Het Q175 mice (*n* = 10 per genotype) were surgically implanted with a wireless radio‐frequency transmitter (ETA‐F20, Data Sciences International, St. Paul, MN). Three of the WT mice and one of the Hom Q175 did not exhibit measureable signals and had to be excluded from the final analysis. Mice were housed in individual cages in the absence of a running wheel. Cages were placed atop telemetry receivers (Data Sciences International) in a light and temperature‐controlled chamber. Standard rodent chow was provided ad libitum. Data collection began 2‐weeks postsurgery, to allow mice to recover in the 12:12 LD cycle. HR is extrapolated from ECG waveforms using the RR interval. All measurements were made in both LD and DD conditions at 3 months (young) and 9 months (middle age). At each age, the recordings took 6–8 weeks to complete. Cage activity in DD was also examined by periodogram analysis (ClockLab program, Actimetrics, Wilmette, IL) to estimate the free‐running period.

### HRV

Data were extracted in 20 sec intervals then filtered to remove extreme noise. Remaining valid data segments were averaged into 1 h bins across the 24‐h cycle. The following parameters for time domain analysis were calculated: mean normal‐to‐normal intervals (NN, in msec) and, standard deviation of all NN intervals (SDNN, in msec). For frequency domain analysis, spectra were calculated with a FFT (fast Fourier transform) with 20,000 data points per bin and a periodogram resolution of 8192, three overlapping subseries and a Hamming window (HRV Module, Data Sciences International). Frequency bins were defined as high‐frequency (HF 1.5–5.0 Hz), low‐frequency (LF: 0.2–1.5 Hz) and very low frequency (VLF: 0.01–0.2 Hz), and the power in these bands was calculated (Campen et al. 2005; Thireau et al. 2008). While it is common for LF and HF to be expressed in normalized units, this normalization minimizes the circadian regulation that we are interested in examining (Badilini et al. 2000). Therefore, we reported the raw LF and HF values (mV/Hz).

### Baroreceptor reflex

Methods employed were similar to those described previously (Schroeder et al. 2011). The administration of angiotensin II (ATII) or nitroprusside (NP) triggers acute hypertension or hypotension, respectively. Changes in blood pressure (BP) as a result of administering these drugs should elicit a compensatory response of HR. Differences in the ratio of change in HR to BP (∆HR/∆BP) are suggestive of aberrant signaling to the heart by the ANS. Baroreceptor function was examined during the day (ZT4‐9) in middle age (9 months) mice of each genotype: WT (*n* = 8), Hom Q175 (*n* = 10) and Het Q175 (*n* = 8). These measurements took 4 weeks to complete. The procedure was unsuccessful in some of the mice (WT, *n* = 1; Het Q175, *n* = 2; Hom Q175, *n* = 1) and data could not be used. During the experiment, HR was determined from the R‐R interval of the ECG. Mice were anesthetized and both femoral arteries were catheterized, whereby BP measurements were collected from one artery and drugs were administered through the other artery. Following catheterization, isoflurane levels were decreased to between 1 and 1.5% and the mice were allowed to rest for at least 10 min. Baseline BP and HR were then recorded. AT II (4.0 *μ*g/kg) and NP (40 *μ*g/kg) were administered in sequence to probe the HR response to the changes in BP. Each treatment was followed by a flush of saline (+heparin 3 U/mL) to ensure full delivery of the drugs. As a control, muscarinic (75 *μ*g/kg glycopyrrolate) and *β*‐adrenergic (750 *μ*g/kg propranolol) receptors were blocked before administering another dose of ATII or NP. The absolute value of the maximum change in BP as well as the subsequent directional change in HR was determined. Ratios of these two values (∆HR/∆BP) reflected the magnitude HR change relative to the change in BP that characterizes the sensitivity of the baroreceptor response.

### Echocardiograms

WT (*n* = 12), Q175 Het (*n* = 10) and Q175 Hom (*n* = 10), were group housed and kept in a 12:12 LD cycle with rodent chow provided ad libitum. Mice were examined with echocardiograms at 3, 6, 9, 12 months of age. After the final measurement, the mice were perfused and morphological and histological measurements of the heart were taken. Echocardiograms were measured using a Siemens Acuson Sequoia C256 instrument equipped with a 15L8 15 MHz probe (Siemens Medical Solutions, Mountain View, CA) as previously described (Schroeder et al. 2016). Briefly, two‐dimensional, M‐mode echocardiography and spectral Doppler images enabled measurement of heart dimension and function (Left ventricle (Lv) Mass), end‐diastolic dimension (EDD), end‐systolic dimension (ESD), posterior wall thickness (PWT), ventricular septal thickness (VST), ratio of the early (E) to late (A) ventricular filling velocities (E/A ratio), fractional shortening (FS%), and Lv Ejection Fraction (Lv EF).

### Morphometry and histology

Following the longitudinal echocardiogram measurements, animals were deeply anaesthetized using isoflurane and perfused with phosphate‐buffered saline (PBS, pH 7.4) with heparin (2 units/ml, Henry Schein, Melville, NY) followed by 4% (w/v) paraformaldehyde (Sigma‐Aldrich) in PBS (pH 7.4). A motorized pump was used to deliver the solutions in order to control and maintain similar pressure between animals. Hearts were embedded in paraffin and processed for Masson's Trichrome staining as previously reported (Schroeder et al. 2016). Two mid‐ventricular‐stained cross sections were used to obtain morphometric measurements and to estimate the number of fibrotic areas in WT (*n* = 5), Hom and Het Q175 mice (both *n* = 6 per genotype). Images of the mid‐ventricular cross sections from each heart were acquired on a Zeiss Stereomicroscope (Stemi SV 11 Apo) equipped with an Axiocam using the AxioVision software (Carl Zeiss). Measurements were performed by two observers masked to the genotype of the mice using the Zeiss Axiovision software. The thickness of the left ventricular wall was measured in three different nonseptal parts to obtain an average thickness. Values from the two sections were averaged. Fibrotic areas, suggestive of necrotic areas, that is, infarcts, were visually identified and counted by three observers masked to the genotype on a Zeiss Axioskop (Carl Zeiss, Pleasanton, CA). To determine which chamber or part of the interventricular septum was more affected, the locations of the infarctions in the heart were also annotated.

For wheat germ agglutinin (WGA) staining, deparaffinized sections were rehydrated through an alcohol gradient starting at 95% ethanol to 50%. Heat‐induced antigen retrieval was performed using 10 mmol/L Citrate buffer (pH 6.0), followed by blocking in 10% BSA/PBS for 1 h. Slides were incubated with Alexa Fluor 594‐WGA (50 *μ*g/mL, 10 min, Invitrogen) in PBS and mounted using ProLong Gold (Invitrogen, Carlsbad, CA). Confocal images of stained sections at the levels of the papillary muscles were captured, using a confocal scanning microscope (Nikon, Melville, NY) and the cross‐sectional area of the cardiomyocytes was measured using the NIS Elements (Nikon) software (50‐100 cells from 6 to 8 areas per mouse heart, *n* = 6 per genotype).

### Statistical analysis

For most of our experiments, two‐way analysis of variance (ANOVA) was used to determine statistical significance with genotype (WT, Het Q175, Hom Q175) and time (either ZT or CT) as the two factors. If the data did not exhibit a normal distribution, a two‐way ANOVA on ranks was used. Post hoc *t*‐test with Bonferroni's corrections were applied to determine significant differences between genotypes at each of the hourly bins (ZT or CT). In the cases in which we only obtained the measurements at one age (baroreceptor, histological measurements), we used a one‐way ANOVA followed by Bonferroni's multiple comparison test or a one‐way ANOVA on ranks. Correlations between activity and heart rate were examined by applying the Spearman correlation analysis to 21,700 contiguous, 20 sec, time‐matched, heart rate and activity bins per mouse at two ages (3 months, 9 months). *R*‐values were then compared between groups using a two‐way ANOVA. For a comparison of the number of infarcts, we used a Kruskal–Wallis ANOVA followed by Dunn's multiple comparison test. These statistical analyses were performed using the SigmaStat (San Jose, CA) or Prism 5 (GraphPad Software, San Diego, CA) statistical software. Values are reported as the mean ± Standard Error of the Mean (SEM).

## Results

**Figure 1:**
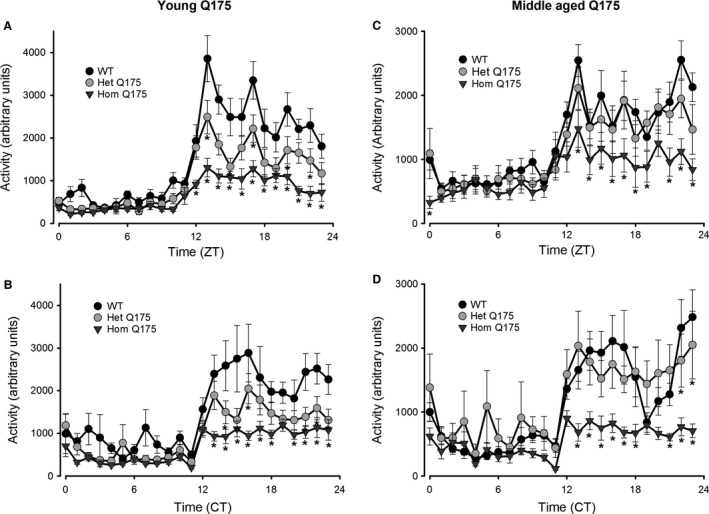
Q175 mice exhibited reduced amplitude of the diurnal and circadian activity rhythms as measured by telemetry starting at 3 months of age (WT,* n* = 8; Het, *n* = 10; Hom, *n* = 9 for all of the telemetry measurements). All of the mice exhibited significant activity rhythms in LD and DD conditions. The activity levels during the night were significantly altered by the Q175 mutation in the young mutants. (A) LD conditions: activity was unaltered during the day but significantly reduced throughout the night in the Hom and Het Q175 compared to WT. (B) DD conditions: activity was significantly reduced throughout the subjective night in the Hom and Het Q175 compared to WT. (C) By middle age, activity was significantly reduced only in the Hom Q175. The differences in activity between Het Q175 and WT were no longer significant as the WT mice began exhibiting the typical age‐related decline. (D) In DD conditions, the middle aged Hom Q175 exhibited greatly reduced activity during the night. Please see Table 1 for results of statistical tests. Asterisks indicate *P *<* *0.05 between the genotypes at each of the hourly bins (ZT or CT).

The aim of this study was to determine if autonomic dysfunction can be detected early in disease progression in the Q175 model and whether the circadian cycle influences it. We used telemetry to assess Q175 rhythms in activity, CBT and HR in young (3 months) and middle aged (9 months) mice. All of the mice exhibited significant day‐night differences under 12:12 LD and subjective day‐night under DD conditions (Table 1). Under LD conditions, activity was unaltered during the day but significantly reduced throughout the night in the Hom and Het Q175 compared to WT (Fig. 1A). These differences in activity were also seen when the mice were held in DD conditions, and emerged in the subjective night between CT 13 and 23 (Fig. 1B). By middle age, there were significant differences in the activity rhythms in LD and DD (Table 1). Under both conditions, the Hom Q175 exhibited greatly reduced activity during the night, while the Het Q175 were not different from WT mice, except for a reduction in activity near the end of the subjective night (Fig. 1C, D). In each of these conditions, the amplitude of the activity rhythms was reduced in the mutants, while the period of the circadian rhythms did not change with the genotype [e.g., WT = 23.86 ± 0.05; Het Q175 = 23.81 ± 0.05; Hom Q175 = 23.86 ± 0.03]. Overall, the Q175 mutants displayed significantly reduced activity levels but these effects were significant only during the night (LD) and subjective night (DD).

**Table 1:** Two‐way ANOVA was used to determine the impact of the Q175 mutation on activity, CBT, resting HR, active HR, and HRV as measured by telemetry in LD and DD

	Young in LD	Young in DD	Middle age in LD	Middle age in DD
Activity				
Genotype	*F *=* *90.3, *P *<* *0.001	*F *=* *61.2, *P *<* *0.001	*F *=* *32.0, *P *<* *0.001	*F *=* *45.1, *P *<* *0.001
Time	*F *=* *29.0, *P *<* *0.001	*F *=* *15.3, *P *<* *0.001	*F *=* *12.3, *P *<* *0.001	*F *=* *15.4, *P *<* *0.001
Interaction	*F *=* *2.6, *P *<* *0.001	*F *=* *1.4, *P *=* *0.06	*F *=* *0.8, *P *=* *0.71	*F *=* *2.1, *P *<* *0.001
CBT				
Genotype	*F *=* *6.2, *P *=* *0.002	*F *=* *0.8, *P *=* *0.75	*F *=* *22.5, *P *<* *0.001	*F *=* *62.3, *P *<* *0.001
Time	*F *=* *75.6, *P *<* *0.001	*F *=* *112.2, *P *<* *0.001	*F *=* *22.6, *P *<* *0.001	*F *=* *37.0, *P *<* *0.001
Interaction	*F *=* *1.4, *P *=* *0.03	*F *=* *0.8, *P *=* *0.75	*F *=* *0.8, *P *=* *0.85	*F *=* *1.1, *P *=* *0.28
Resting HR				
Genotype	*F *=* *25.5, *P *<* *0.001	*F *=* *14.2, *P *<* *0.001	*F *=* *1.9, *P *=* *0.15	*F *=* *20.1, *P *<* *0.001
Time	*F *=* *13.0, *P *<* *0.001	*F *=* *19.0, *P *<* *0.001	*F *=* *6.8, *P *<* *0.001	*F *=* *14.3, *P *<* *0.001
Interaction	*F *=* *0.6, *P *=* *0.98	*F *=* *0.3, *P *=* *1.00	*F *=* *0.5, *P *=* *0.99	*F *=* *0.7, *P *=* *0.95
Active HR				
Genotype	*F *=* *3.7, *P *=* *0.024	*F *=* *5.1, *P *=* *0.006	*F *=* *13.6, *P *<* *0.001	*F *=* *29.2, *P *<* *0.001
Time	*F *=* *8.6, *P *<* *0.001	*F *=* *5.8, *P *<* *0.001	*F *=* *3.5, *P *<* *0.001	*F *=* *7.1, *P *<* *0.001
Interaction	*F *=* *0.7, *P *=* *0.91	*F *=* *0.8, *P *=* *0.82	*F *=* *0.9, *P *=* *0.66	*F *=* *1.2, *P *=* *0.23

	HRV	HF power	LF power
Genotype	*F *=* *92.4, *P *<* *0.001	*F *=* *6.8, *P *=* *0.001	*F *=* *41.7, *P *<* *0.001
Time	*F *=* *7.3, *P *<* *0.001	*F *=* *11.7 *P *<* *0.001	*F *=* *9.4, *P *<* *0.001
Interaction	*F *=* *0.5, *P *=* *0.99	*F *=* *0.8, *P *=* *0.82	*F *=* *1.3, *P *=* *0.11

**Figure 2:**
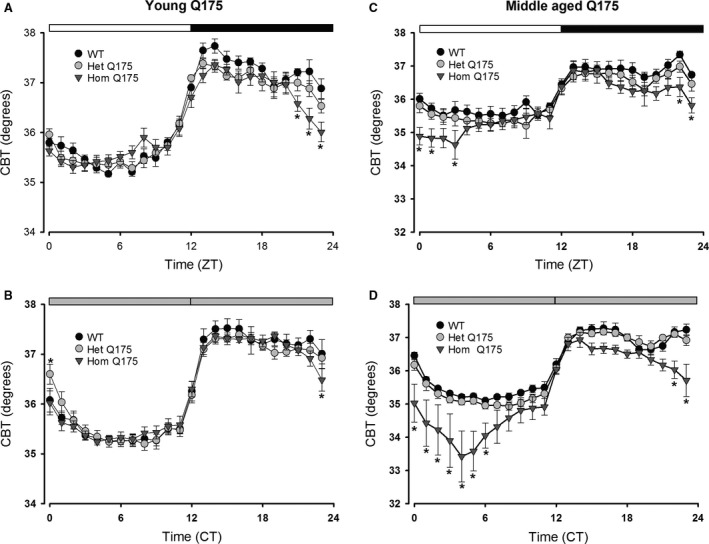
Hom Q175 mice exhibited disrupted rhythms in CBT in middle age. (A, B) The young adult Q175 exhibited a significantly different CBT rhythm in LD and DD conditions. Only a few phases late in the night were impacted. (C, D) By middle age, there were significant differences in the CBT rhythm in LD and DD. The mutant mice exhibited episodes of hypothermia that were most severe in the Hom Q175. Please see Table 1 for results of statistical tests. Asterisks indicate *P *<* *0.05 between the genotypes at each of the hourly bins (ZT or CT).

**Figure 3:**
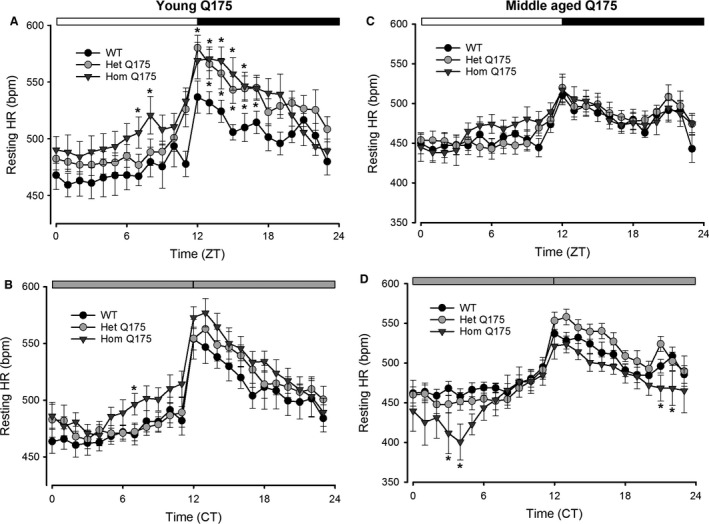
Q175 mice exhibited altered diurnal and circadian resting HR rhythms. (A, B) The young Q175 exhibited significantly different resting HR rhythms under both LD and DD. The effects were most striking under LD conditions where the mutants showed high resting HR. (C, D) By middle age, this tachycardia reversed and we actually saw low resting HR in the Hom Q175 under DD conditions. Please see Table 1 for results of statistical tests. Asterisks indicate *P *<* *0.05 between the genotypes at each of the hourly bins (ZT or CT).

**Figure 4:**
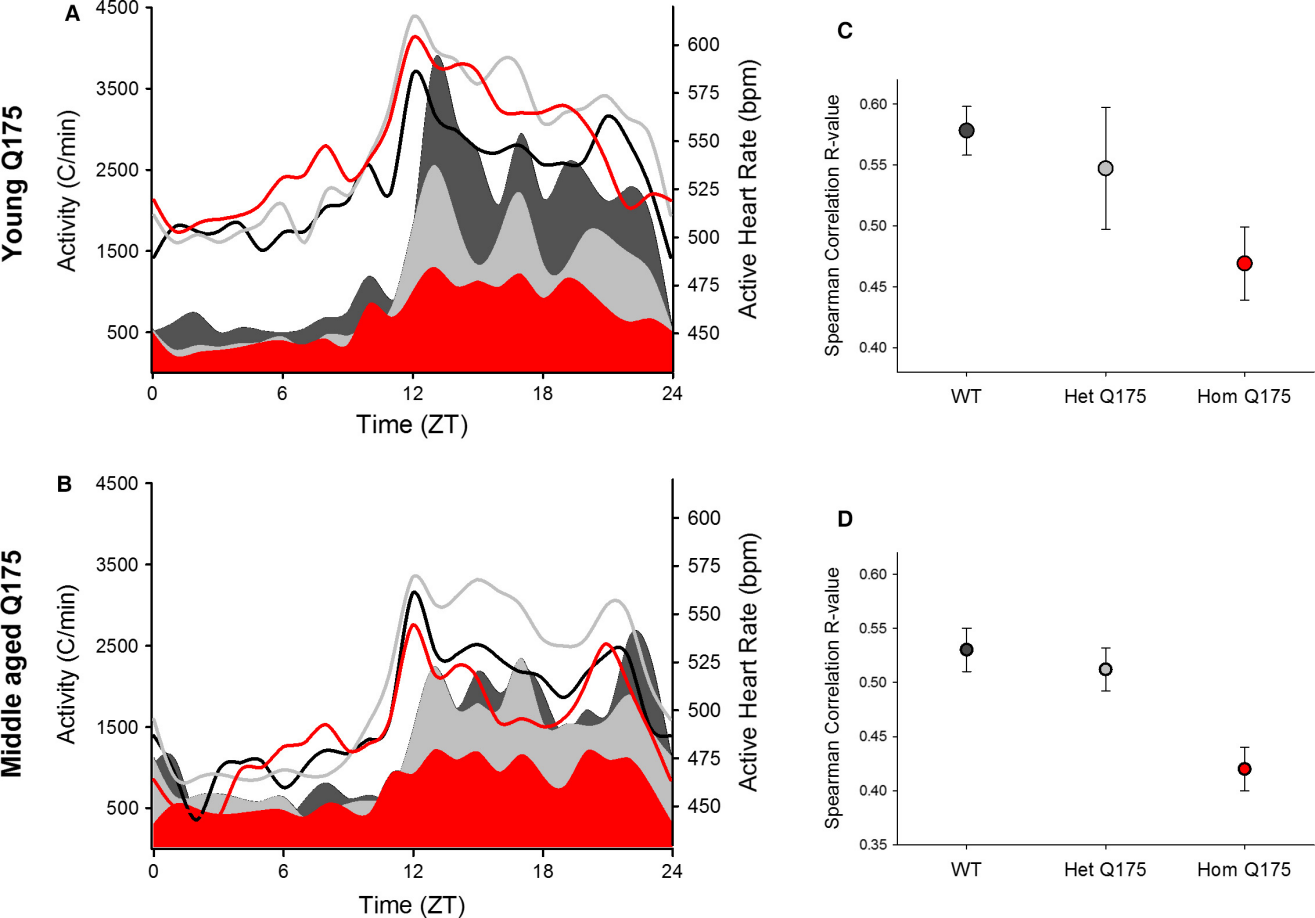
Q175 mice exhibited an abnormal relationship between HR and activity. (A) The young adult Hom Q175 exhibited high HR even while they exhibited low levels of activity. (B) By middle age, the Hom Q175 exhibited lower HR and lower activity in the night. WT data shown with black lines (HR) and fill (activity); the Het Q175 data are shown with gray lines (HR) and fill (activity); the Hom Q175 data are shown with red lines (HR) and fill (activity). C) Q175 homozygotes exhibited significant loss of correlation of HR with activity levels (*P *<* *0.001) in young mice. (D) By middle age, differences are diminished by the effects of aging and the progression of autonomic pathology in the mutants. WT data shown with black lines (HR) and fill (activity); Het Q175 data shown with gray lines (HR) and fill (activity); Hom Q175 data shown with red lines (HR) and fill (activity). WT, wild‐type.

Genotype and Time were the factors.The results of a pair‐wise comparison between the genotypes at each time is shown in Figure 1, 2, 3, 4.

Next, we examined the ANS‐driven rhythms in CBT in young and middle aged mice. The young Q175 mutant mice showed rhythms that were similar to their WT counterparts under LD and DD conditions (Fig. 2A, B). There was a significant effect of genotype in LD but not DD conditions (Table 1). The young Hom Q175 did exhibit significantly reduced body temperature late in the night (Fig. 2A). By middle age, there were significant differences in the CBT rhythm in LD and DD (Table 1). Under both conditions, the Hom Q175 exhibited difficulty maintaining body temperature during the first half of the day (subjective day) and during the late night (subjective night) (Fig. 2C and D). Upon examination of CBT with higher temporal resolution, episodes in which the HD mutants failed to maintain their body temperature emerged. By middle age, these hypothermic episodes were seen in all of the Hom Q175 (8/8) and in almost half (4/10) of the Het Q175 but were absent in WT mice. In the Het Q175, these hypothermic events were short (46 ± 2 min) but, in the Hom Q175, they lasted for more than 5 h (342 ± 116 min). Overall, the Q175 exhibited difficulty in maintaining normal CBT and this regulatory problem emerged early in the Hom Q175 and was more pronounced by middle age.

In addition, we examined the HR when the animals were physically inactive, that is, the resting HR. The young Q175 exhibit abnormally high resting HR under both LD and DD, mostly during the late day and early night (Fig. 3A and B; Table 1). By middle age in DD, this tachycardia reversed and we actually saw low resting HR in the Hom Q175 (Table 1). The low HR in the Hom Q175 occurred primarily during the early subjective day (Fig. 3C and D). The simultaneous recording of HR and activity allows the analysis of HR evoked by movement; hence, we measured HR when the animals were physically active, that is, the active HR. Again, a significant effect of genotype in young animals was found under both LD and DD (Table 1) conditions. The Hom Q175 exhibited a significantly increased active HR during the early day and subjective day (Table 1). Similar results were obtained in middle aged mutants, with a significant effect of genotype under both LD and DD (Table 1) conditions. A significantly increased activity‐evoked HR was observed in the Hom Q175 during the early day and subjective day as well as a reduced correlation between activity and HR (Fig. 4). For example, in WT mice, the Spearman correlation between activity and HR was 0.56 ± 0.03 while in the Hom Q175 the correlation was reduced to 0.42 ± 0.02 (genotype, *F *=* *10.4; *P *<* *0.001). Finally, we compared a number of parameters of the ECG waveform (RR, PR, QRS, QT, QAT, MxdV) in WT and the Q175 mutants under LD conditions (Table 2). Significant differences between the genotypes were seen with RR, QRS, and QAT intervals. In summary, the Hom Q175 exhibited tachycardia during rest and activity with the high HR being most pronounced during the day when HR was normally low. Even in young animals, the ANS‐driven rhythms in HR were disrupted in the Q175 line.

**Table 2:** Electrocardiographic parameters in young Q175 and WT animals held in a LD cycle. ECG values were continuously measured and placed into hourly bins

ECG parameters		WT	Het Q175	Hom Q175	ANOVA genotype
RR (msec)	Day	124 ± 2	121 ± 2	115 ± 2^b^	*F *=* *4.8, *P *=* *0.019
	Night	107 ± 1^a^	106 ± 2^a^	108 ± 2^a^	*F *=* *0.6, *P *=* *0.533
PR (msec)	Day	36 ± 1	34 ± 1	35 ± 1	*F *=* *0.4, *P *=* *0.698
	Night	33 ± 1^a^	32 ± 1^a^	33 ± 1^a^	*H *=* *0.3, *P *=* *0.858
QRS (msec)	Day	13.2 ± 0.1	13.5 ± 0.2	13.2 ± 0.2	*H *=* *3.8, *P *=* *0.147
	Night	12.8 ± 0.1^a^	13.1 ± 0.2^a^	12.6 ± 0.1^a^	*H *=* *5.0, *P *=* *0.080
QR (msec)	Day	6.5 ± 0.1	6.6 ± 0.1	6.6 ± 0.1	*F *=* *0.3, *P *=* *0.724
	Night	6.3 ± 0.1^a^	6.4 ± 0.1^a^	6.3 ± 0.1^a^	*F *=* *0.4, *P *=* *0.662
QAT (msec)	Day	31 ± 6	31 ± 5	20 ± 1^b^	*H *=* *8.6, *P *=* *0.013
	Night	34 ± 6^a^	30 ± 4	21 ± 1^b^	*F *=* *3.9, *P *=* *0.036
ST (msec)	Day	51 ± 3	56 ± 3	55 ± 3	*H *=* *2.9, *P *=* *0.236
	Night	52 ± 4	52 ± 3^a^	52 ± 3^a^	*H *=* *0.2, P = 0.871
MxdV (mV/msec)	Day	274 ± 22	348 ± 27	329 ± 41	*F *=* *1.4, *P *=* *0.251
	Night	348 ± 28^a^	411 ± 36^a^	394 ± 43^a^	*F *=* *0.8, *P *=* *0.468

One‐way ANOVA was used to assess differences between the genotypes in the day or night. A paired *t*‐test was used to access day/night differences within a genotype.

a *P* < 0.05 day versus night within a genotype.

b *P* < 0.05 significant difference compared to WT.

**Figure 5:**
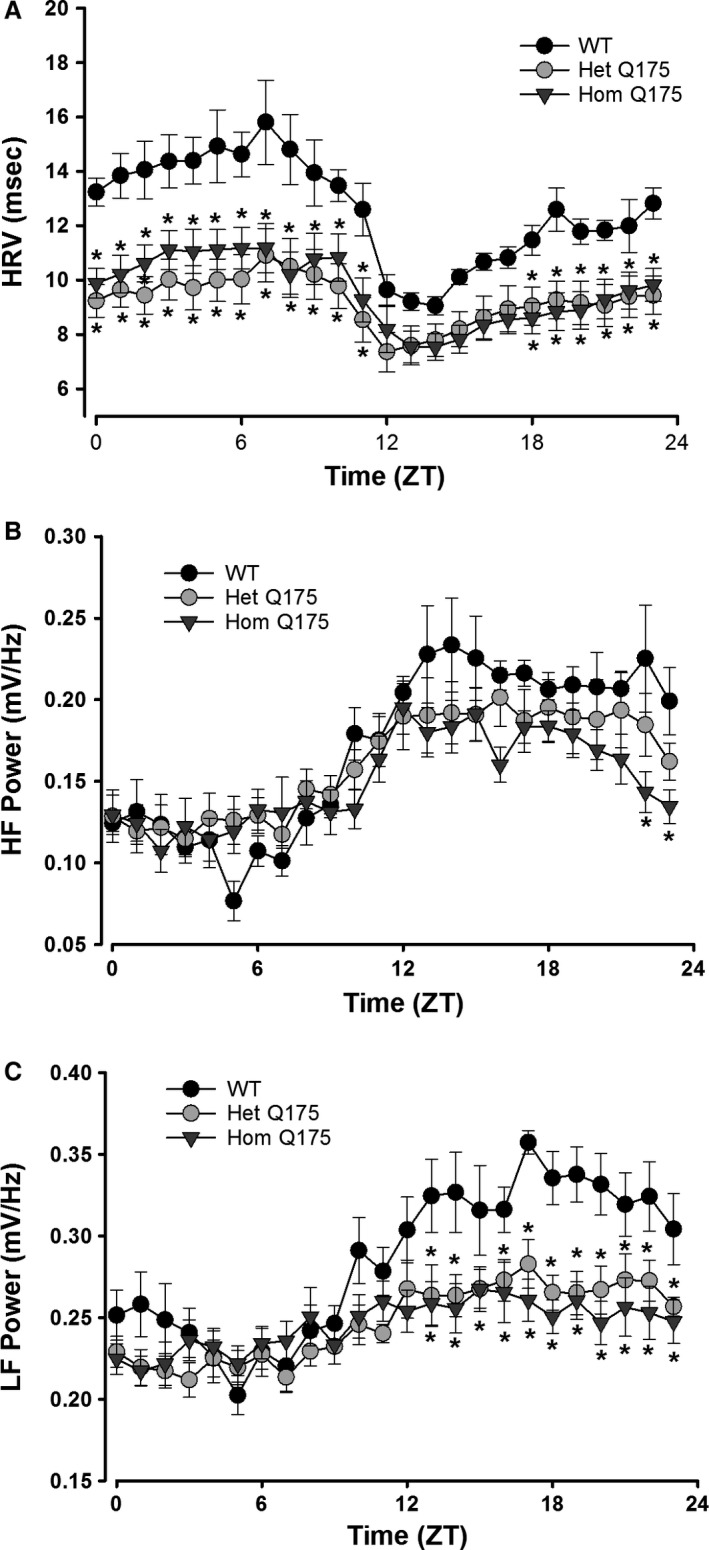
Q175 mice exhibited low HRV as measured at 3 months. (A) The young Q175 (Hom, Het) exhibited significantly reduced HRV. Most phases of the daily cycle were impacted. (B) The HF domain (1.5–5.0 Hz) was largely unaltered except at 2 phases late in the night. (C) The LF domain (0.2–1.5 Hz) was reduced in the mutants (Hom, Het) throughout the night. Please see Table 1 for results of statistical tests. Asterisks indicate *P *<* *0.05 between the genotypes at each of the hourly bins (ZT or CT).

To further explore autonomic dysfunction in the Q175 line, we measured HRV throughout the 24‐hr cycle. HRV measures the variability in the time between individual heartbeats and reflects the balance of the sympathetic and vagal inputs regulating heart rate. High HRV is associated with cardiovascular health, while low HRV is a sign of poor cardiovascular function. We observed high HRV during the day and lower at night (Fig. 5A) in WT mice. Conversely, the HRV was abnormally low in the HD mutants (Hom, Het) and this damping was largest during the sleep phases. HRV measures were separated into low‐frequency (LF: 0.2–1.5 Hz) and high‐frequency (HF, 1.5–5.0 Hz) bands, which are commonly used to quantify parasympathetic and sympathetic regulation, respectively. In WT mice, the power of both the LF and HF bands exhibited a daily rhythm which peaked during the night (Fig. 5B and C). The power of the HF band was minimally impacted with the young Hom Q175 exhibiting significant differences only in the late night (Fig. 5B). In contrast, the power of the LF band was greatly impacted throughout the night in the mutants (Fig. 5C). These results demonstrate that the temporal patterning as well as the overall level of autonomic regulation of the cardiovascular system is compromised in the Q175 mice.

**Figure 6:**
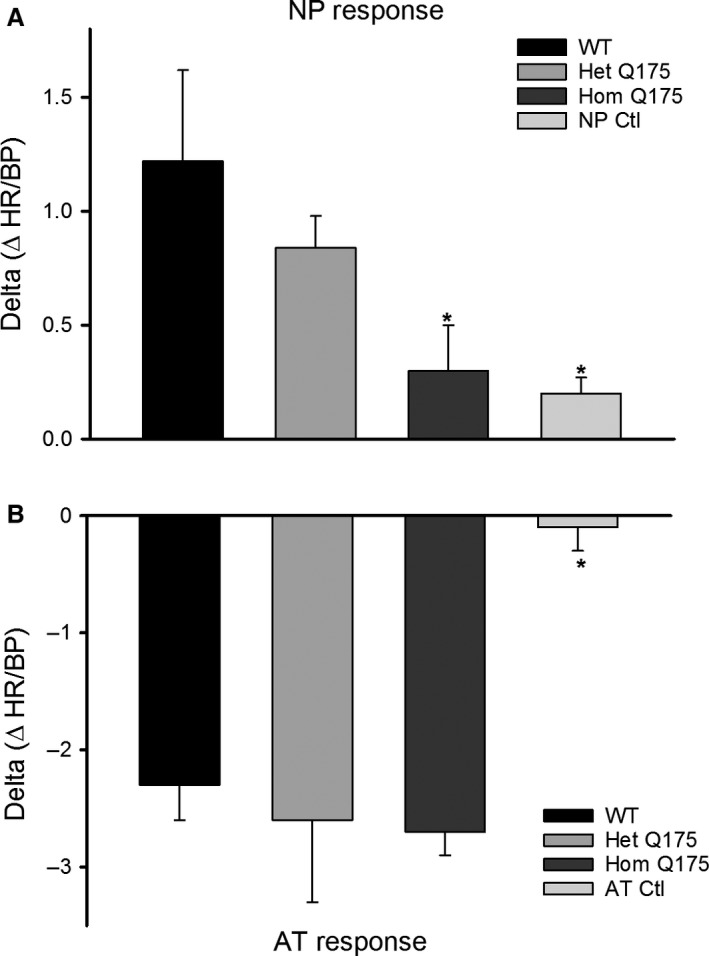
Baroreceptor evaluations were used to determine the autonomic responsivity to blood pressure perturbations (WT,* n* = 7; Het, *n* = 6; Hom, *n* = 9). As expected, the WT mice had normal reflex responses. (A) When the blood pressure was transiently lowered with nitroprusside (NP, 40 *μ*g/kg), the heart rate was elevated. (B) When the blood pressure was transiently increased with angiotensin II (AT, 4 *μ*g/kg), the heart rate dropped in compensation. The impact of ATII and NP were blocked by pretreatment with sympathetic and parasympathetic receptor antagonists (750 *μ*g/kg propranolol, 75 *μ*g/kg glycopyrrolate). The Hom Q175 mice had very blunted HR responses to NP (*n* = 9). While some of the Het Q175 (2 out of 6 animals) showed a greatly reduced response to the NP injection, the overall NP‐response was not significantly different from WT. WT, wild‐type, ATII, administration of angiotensin II.

The baroreceptor reflex is a rapid homeostatic mechanism by which pertubations in blood pressure are countered by change in HR mediated by the ANS. We performed baroreceptor evaluations to determine the autonomic responsivity to drug‐induced changes in BP (Fig. 6A and B). As expected, the middle‐aged WT mice had normal reflex responses, that is, when the BP was transiently increased with angiotensin II (ATII, 4 *μ*g/kg), the heart rate dropped in compensation, and when the BP was transiently lowered with nitroprusside (NP, 40 *μ*g/kg), the heart rate was elevated. The effects of ATII and NP were completely blocked by pretreatment with sympathetic and parasympathetic receptor antagonists (750 *μ*g/kg propranolol, 75 *μ*g/kg glycopyrrolate)(Fig. 6). Interestingly, the Hom Q175 mice had very blunted HR responses to NP (one‐way ANOVA on ranks: *H *=* *11.3, *P *=* *0.004). While some of the Het Q175 (2/6) showed a greatly reduced response to the NP injection, the overall NP‐response was not significantly different from the WT (Fig. 6A). There were no significant changes in response to ATII injection although we did observe an abnormal instability in the Hom Q175 HR (Fig. 6B). Systolic BP did not significantly vary with genotype (WT: 83 ± 4 mm Hg; Het Q175: 76 ± 4; Hom Q175: 71 ± 3; *F *=* *2.8; *P *=* *0.08). These data provide further evidence of diminished autonomic function, especially of the sympathetic branch.

**Figure 7:**
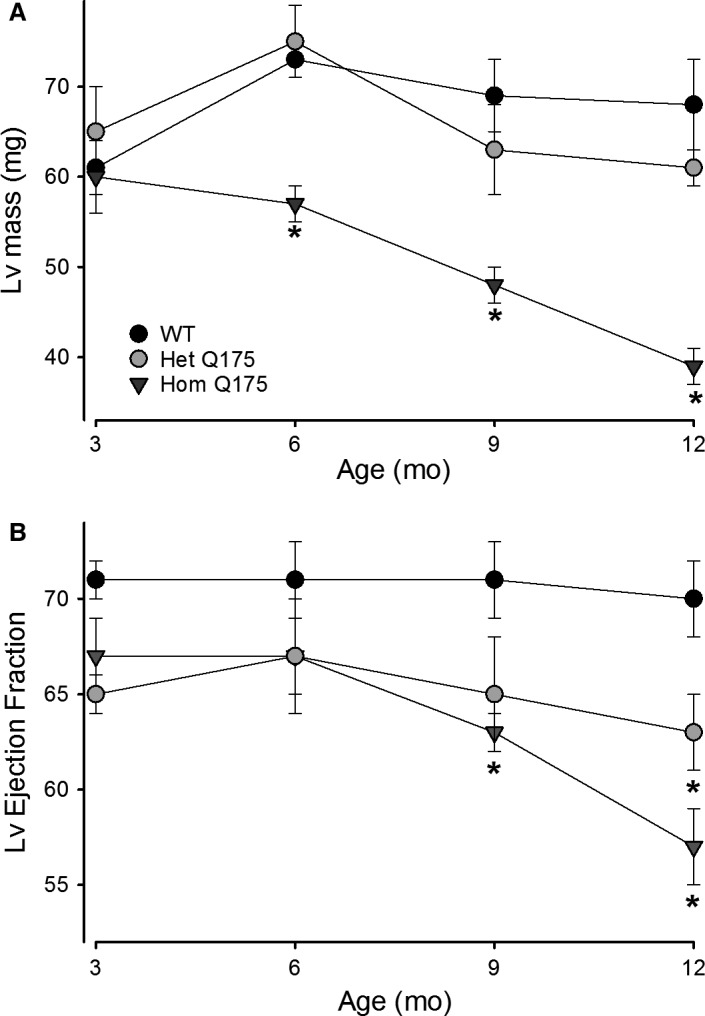
The age‐dependent progression in heart dysfunction was evaluated in a separate cohort of mice using echocardiograms starting at 3 months of age and progressing to 12 months (WT,* n* = 12; Het, *n* = 10; Hom, *n* = 10). Both cardiac structural (Lv mass, EDD, ESD) and functional (Lv % FS, E/A ratio, and Lv EF) deficits were observed and exhibited significant effects of age and genotype (Table 4).

The age‐dependent progression in heart dysfunction was evaluated in a separate cohort of mice using echocardiograms beginning at 3 months of age and progressing to 12 months (Fig. 7; Table 3). Both the cardiac structure [Lv mass, EDD, ESD) and function (FS%, E/A ratio, Lv EF) were significantly influenced by age and genotype (Table 4). For example, the Lv mass of the Hom Q175 was significantly smaller compared to WT and Het Q175 by 6 months of age (Fig. 7A). Cardiac function, measured by Lv EF, was significantly diminished in the Hom Q175 beginning at 9 months. Both the Hom and Het Q175 exhibited a reduction in this parameter compared to WT at 12 months (Fig. 7B). By 9 months of age, the hearts of Hom Q175 mice were smaller and functionally depressed compared to WTs.

**Table 3:** Echocardiographic parameters in Q175 and WT animals beginning at 3 months of age

	WT	Het Q175	Hom Q175
Age (months)	3	3	3
Lv mass (mg)	61 ± 3	65 ± 5	60 ± 4
EDD (mm)	4.1 ± 0.1	3.9 ± 0.1	3.8 ± 0.1
ESD (mm)	2.7 ± 0.1	2.7 ± 0.1	2.6 ± 0.1
Ao‐ET (msec)	50 ± 1	52 ± 2	54 ± 2
FS (%)	35 ± 1	33 ± 1	32 ± 1
E/A	1.7 ± 0.1	1.7 ± 0.1	1.8 ± 0.1
Lv EF	71 ± 1	65 ± 1	67 ± 2
Age (months)	6	6	6
Lv mass (mg)	72 ± 3	74 ± 4	57 ± 2
EDD (mm)	4.2 ± 0.1	4.2 ± 0.1	3.8 ± 0.1
ESD (mm)	2.7 ± 0.1	2.8 ± 0.1	2.5 ± 0.1
Ao‐ET (msec)	52 ± 1	52 ± 1	58 ± 2^a^
FS (%)	35 ± 2	33 ± 2	34 ± 1
E/A	1.8 ± 0.1	1.8 ± 0.1	1.7 ± 0.1
Lv EF	71 ± 2	67 ± 2.3	67 ± 3
Age (months)	9	9	9
Lv mass (mg)	69 ± 4	63 ± 5	48 ± 2^a^
EDD (mm)	4.2 ± 0.1	4.0 ± 0.1	3.4 ± 0.1
ESD (mm)	2.7 ± 0.1	2.8 ± 0.1	2.5 ± 0.1
Ao‐ET (msec)	48 ± 1	53 ± 1	54 ± 2^a^
FS (%)	36 ± 2	28 ± 2^a^	27 ± 1^a^
E/A	1.7 ± 0.1	1.6 ± 0.1	1.5 ± 0.1^a^
Lv EF	71 ± 2	65 ± 3	63 ± 1^a^
Age (months)	12	12	12
Lv mass (mg)	68 ± 5	61 ± 2^a^	39 ± 2^a^
EDD (mm)	4.1 ± 0.1	3.8 ± 0.1	3.2 ± 0.1
ESD (mm)	2.7 ± 0.1	2.8 ± 0.1	2.4 ± 0.1
Ao‐ET (msec)	50 ± 1	52 ± 2	57 ± 1^a^
FS (%)	34 ± 2	26 ± 2^a^	25 ± 1^a^
E/A	1.9 ± 0.1	1.5 ± 0.1^a^	1.5 ± 0.1^a^
Lv EF	70 ± 2	64 ± 3^a^	58 ± 2^a^

Two‐dimensional, M‐mode echocardiography and spectral Doppler images enabled measurement of heart dimension and function including Left ventricle mass (Lv mass), end‐diastolic dimension (EDD), end‐systolic dimension (ESD), posterior wall thickness (PWT), ventricular septal thickness (VST), aorta ejection time (Ao‐ET), ratio of the early (E) to late (A) ventricular filling velocities (E/A ratio), fractional shortening (FS%), Lv Ejection Fraction (Lv EF).

a *P* < 0.05 significant difference compared to WT.

**Table 4:** Two‐way ANOVA was used to determine whether each of the echocardiographic parameters measured in Q175 and WT mice were significantly different

	Lv mass (mg)	EDD (mm)	ESD (mm)	Ao‐ET (ms)	FS (%)	Lv EF
Genotype	*F *=* *23.1, *P *<* *0.001	*F *=* *34.1, *P *<* *0.001	*F *=* *6.1, *P *=* *0.003	*F *=* *21.6, *P *<* *0.001	*F *=* *8.6, *P *<* *0.001	*F *=* *10.6, *P *<* *0.001
Age	*F *=* *6.4, *P *<* *0.001	*F *=* *6.6, *P *<* *0.001	*F *=* *0.2, *P *=* *0.887	*F *=* *3.1, *P *=* *0.031	*F *=* *4.5, *P *=* *0.005	*F *=* *5.0, *P *=* *0.003
Interaction	*F *=* *3.2, *P *=* *0.006	*F *=* *2.9, *P *=* *0.01	*F *=* *0.5, *P *=* *0.780	*F *=* *0.5, *P *=* *0.787	*F *=* *1.6, *P *=* *0.156	*F *=* *1.5, *P *=* *0.197

Genotype and age were the two factors. Measurements were made when the mice were 3, 6, 9, and 12 months of age.

**Figure 8:**
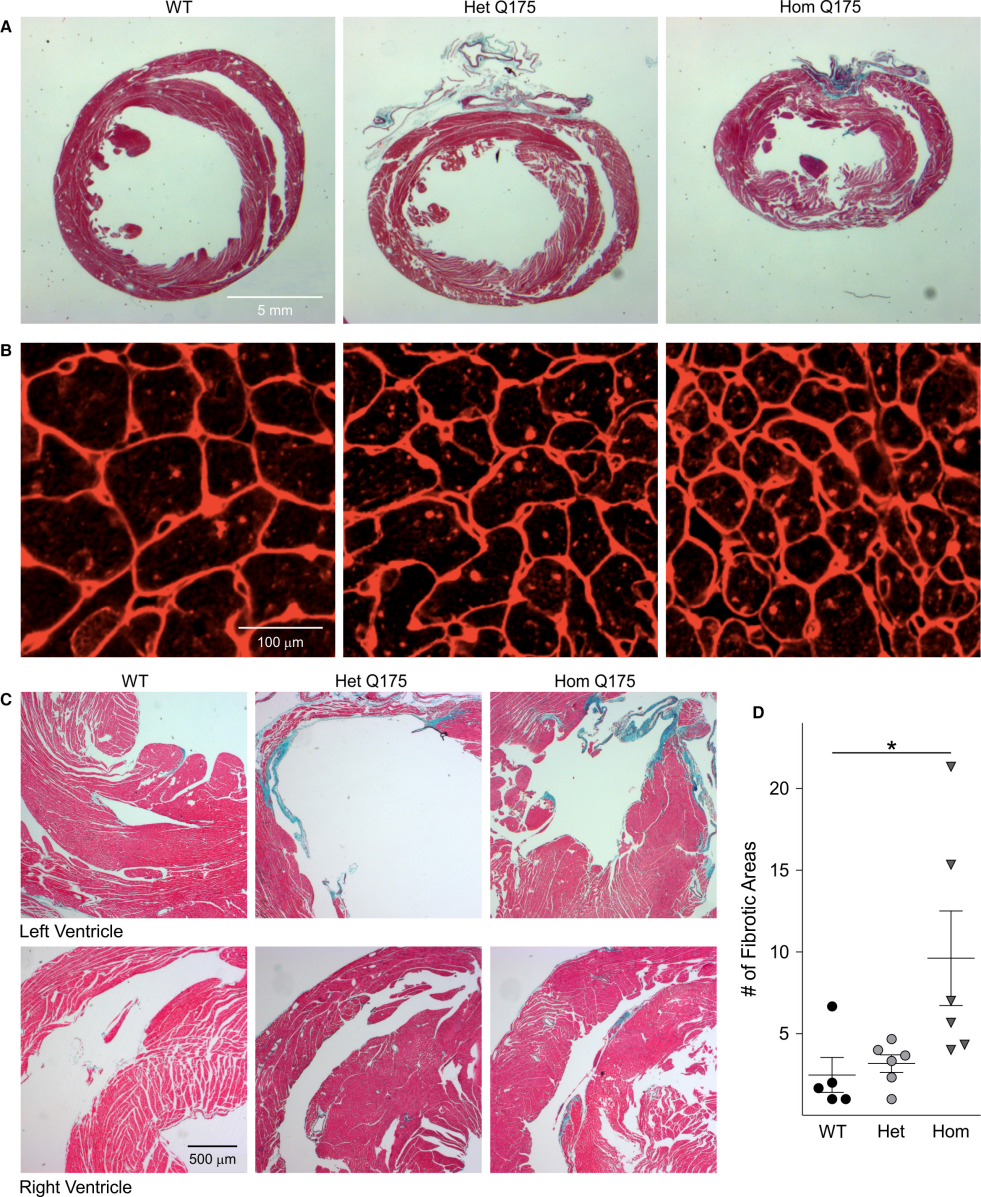
Histological analyses indicated heart pathology in the Q175 mouse. (A) Representative images of Masson's trichrome stained hearts of each genotype showing that the gross dimensions of the Hom Q175 heart were strikingly smaller. (B) Mutants had reduced cardiomyocyte size as well as altered cytoarchitecture as shown by WGA staining. (C) Masson's trichrome stain revealed greatly increased incidence of fibrotic lesions in Q175 Hom hearts. (D) These infarcts were present in each genotype, but were more common (about 3–4 folds) in both ventricles and anteriorly in the interventricular septum of the Hom Q175. Please see Table 5 and 6 for the results of statistical tests. Data are shown as the Mean ± SEM (*n* = 5–6 animals/genotype). *P <* *0.05. The comparison between the number of infarcts was made with Kruskal–Wallis ANOVA followed by Dunn's Multiple Comparison Test.

We observed a significant reduction in both the body (−25%) and heart (−18%) weight in the Hom Q175 as compared to WT (Table 5). Therefore, at the end of the echocardiogram measurements, histological analysis of the hearts was performed to further characterize these abnormalities. The total area of coronal heart sections at the mid‐ventricular level in the Hom Q175 was 33% and 23% smaller than that of WT and Het Q175, respectively (Table 5, Fig 8A). In agreement with the echocardiogram data, the Lv lumen cross sectional area (CSA) (54%) and circumference (23%) were greatly reduced in the Hom Q175 (Table 5). No differences were found in the thickness of the Lv wall or the interventricular septum. The dimensions of the Het Q175 hearts were not significantly different from WT (Table 5). Next, we measured the CSA of individual cardiomyocytes stained with WGA (Fig. 8B). While the WT cardiomyocytes exhibited a medium CSA of 204 ± 11 *μ*m^2^, the CSA was 151 ± 4 *μ*m^2^ and 95 ± 8 *μ*m^2^ in the Het and Hom Q175, respectively (ANOVA on ranks, *H *=* *15.15, *P *<* *0.0001). This dramatic reduction in size (53%) of the CSA of the cardiomyocyte in the Hom hearts was accompanied by an apparent disorganized cytoarchitecture of the myocardium (Fig. 8B). Finally, histopathological analysis of the heart with Masson's trichrome stain revealed local pockets of fibrosis where the cardiomyocytes appeared to be replaced by scar tissue (Fig. 8C). These areas, an indication of small necrotic lesions or infarcts, were present in the heart of all three genotypes with an average of 9.6 infarcts/section in the Hom Q175 vs 2–3 infarcts/section in the WT and Het Q175 (Fig. 8D). Small fibrotic areas were occasionally seen in all the WT mice examined, frequently on the edge of the papillary formations in the Lv (Fig. 8C). In the mutants, the fibrotic pockets were seen in the ventricular wall of both the left and right ventricles, some of which were extensive. Interestingly, in the Hom Q175, these lesions were most commonly seen in the anterior part of the interventricular septum near the anterior groove, suggesting disruption of the blood supply to the heart (Fig. 8C,D).

**Table 5:** Morphological measurements in WT and Q175 mutants. were performed in heart coronal sections at the mid‐ventricular level chosen based on the visual presence of papillary muscles in the left ventricle (Lv)

	WT	Het Q175	Hom Q175	One‐way ANOVA
Body Weight (g)	28 ± 0.7	26 ± 0.4	21 ± 0.4^a^	*F* = 64.5, *P* < 0.001
Heart Weight (mg)	153 ± 4	151 ± 1	125 ± 2^a^	*H* = 19.4, *P* < 0.001
Total heart cross sectional area (mm^2^)	31.2 ± 1.3	27.1 ± 1.7	20.7 ± 0.7^d^^,^ f	*F* = 16; *P* = 0.0002
Circumference/perimeter (mm)	20.0 ± 0.3	18.8 ± 0.6	17.2 ± 0.5^c^	*F* = 7.9; *P* = 0.0049
Interventricular septum thickness (mm)	1.1 ± 0.1	1.1 ± 0.1	1.0 ± 0.06	*F* = 0.6, *P* = 0.561
Lv thickness (mm)	1.09 ± 0.06	1.09 ± 0.07	1.12 ± 0.05	*F* = 0.08, *P* = 0.917
Lv lumen cross sectional area (mm^2^)	8.9 ± 1.0	6.8 ± 0. 6	4.0 ± 0.3^d^^,^ e	*F* = 13.7, *P* = 0.0005
Lv lumen circumference/perimeter (mm)	10.7 ± 0.6	10.0 ± 0.6	8.2 ± 0.5^b^	*F *= 5.26; *P = 0.0198*

Since the Lv were not perfectly round, we refer to the lumen measurements as circumference/perimeter. Values are presented as the Mean ± SEM. WT, *n* = 5; Het Q175, *n* = 6; Hom Q175, *n* = 6).

a *P* < 0.001 versus WT.

b *P* < 0.05.

c *P* < 0.005.

d *P* < 0.0005 versus WT.

e *P* < 0.05.

f *P* < 0.005 versus Het one‐way ANOVA followed by Bonferroni's multiple comparison test.

## Discussion

In this study, we sought to test the dual hypotheses that cardiovascular dysautonomia can be detected early in disease progression in the Q175 model and that this dysfunction varies with the daily cycle. We found overwhelming evidence for autonomic dysfunction including blunted daily rhythms in HR and CBT, reduced HRV, and almost a complete failure of the sympathetic arm of the ANS during the baroreceptor challenge. Importantly, several aspects of the Q175 phenotype were found to vary with a diurnal and circadian phase dependence. For example, activity measurements solely during the day would not have uncovered deficits at the young ages and HRV deficits were more prominent during the day but it would have been difficult to detect during the night. Hence, the time of measurement and the circadian cycle has to be considered as a critical factor in the expression of HD symptoms. The Q175 mouse model of HD exhibits cardiovascular symptoms comparable to those seen in HD patients with a prominent sympathetic dysfunction during the resting phase.

Q175 (Hom, Het) mice showed deficits in both diurnal and circadian activity rhythms (Fig. 1) with the Hom Q175 exhibiting striking deficiencies as young adult mice (3 months). A variety of studies have now demonstrated that four distinct mouse models of HD all exhibit a progressive and rapid breakdown of the circadian rest/activity cycle (Morton et al. 2005; Bode et al. 2009; Kudo et al. 2011; Oakeshott et al. 2011; Loh et al. 2013). Rhythms in locomotor activity are generated in the central circadian clock or SCN. The circuitry by which the SCN regulates locomotor patterns is not clearly established but there is evidence for both neural and hormonal SCN outputs (Li et al. 2012). At this point, we have no evidence that degeneration in the central clock underlies the observed behavioral disruption, although, prior work has shown cell loss in the SCN of HD patients (van Wamelen et al. 2013). We have previously shown that middle‐age Q175 mice show severe circadian dysfunction with no changes in the size of the Nissl‐defined SCN (Loh et al. 2013) or in the phasing of expression of the clock gene *Period2*. In agreement, here, we report no changes in the free‐running period of the circadian system. Together this evidence makes it unlikely that the molecular clockwork that drives circadian rhythms is altered, but indicates that the outputs of the SCN clock are certainly impacted. Reduced SCN neural activity was also reported in the BACHD model (Kudo et al. 2011; Kuljis et al. 2016). Thus, a weakening of the output from the SCN provides the most likely explanation for the reduced amplitude of the circadian rhythm in locomotor activity observed in this study.

Q175 (Hom, Het) mice showed deficits in both diurnal and circadian activity rhythms in CBT (Fig. 2) and HR (Fig. 3) with the deficits in HR occurring in young adults while the deficits in CBT were not observed until middle age. The circadian rhythms in CBT and HR are independent of locomotor activity but dependent on an intact SCN (Ruby et al. 2002; Saleh and Winget 1977; Scheer et al. 2005; Stephan and Nunez 1977; Warren et al. 1994; Witte et al. 1998). The SCN projects to the dorsal subparaventricular zone (Leak and Moore 2001; Deurveilher and Semba 2003), which is necessary for driving circadian rhythms of body temperature (Lu et al. 2001). In fact, as reported by Refinetti et al. (1994), just a small number of SCN neurons is sufficient to maintain circadian rhythm in CBT. Thus, the lack of an impact on the CBT rhythm suggests that at least some of the SCN outputs are intact in the young Q175 mice. Young Hom Q175 exhibited highly pronounced tachycardia during their normal sleep time, with high HR and a reduced amplitude in the HR rhythm (Fig. 3). In addition, the normal strong correlation between activity and HR, which is mediated by the ANS, was dramatically reduced in the mutants (Fig. 4). These changes suggest that ANS disruption occurs early in disease progression in the Q175 line. In agreement, we also saw evidence in the BACHD of the circadian system failing to lower blood pressure during sleep (Schroeder et al. 2011). Similarly, R6/1 mice have a higher HR than WT littermates in young but not older mutant mice (Kiriazis et al. 2012). However, in two other models (R6/2 and HdhQ150 lines), the HR was significantly reduced in symptomatic mice (Mielcarek et al. 2014).

HRV is a measure of variation in the beat‐to‐beat (R‐R) interval that reflects the dynamic balance of sympathetic and parasympathetic control of heart function. In WT mice, HRV displayed a robust diurnal and circadian rhythm consistent with circadian regulation of the ANS (Fig. 5). Both the LF and HF domains of the HRV exhibited robust daily rhythms. In the Q175 line, the HRV was low in young mutants (Hom, Het) and this reduction was largest during the rest phase (Fig. 5A). The power in the LF domain was significantly reduced in the young mutants with the biggest effects during the night (Fig. 5B). Traditionally, the LF is viewed as a measure of regulation by the sympathetic branch of the ANS, although it is more likely a general index of ANS function (Burr 2007; Heathers 2014). Thus, our data clearly indicate that the autonomic outflow is disrupted in the HD mutants. Autonomic dysfunction is likely responsible for the low HRV as well as the elevated HR during the rest phase (daytime) in Q175 mice. A similar reduction in HRV was previously observed in the BACHD line (Kudo et al. 2011), and evidence of disrupted ANS leading to an unstable heart beat as well as elevated levels of the sympathetic transmitter (norepinephrine) were reported in the R6/1 model (Kiriazis et al. 2012). It is also possible that postsynaptic receptors are altered, as suggested by a recent study showing that acetylcholine receptors (measured by *α*‐bungarotoxin binding) are reduced in the diaphragm of BACHD mice (de Aragão et al. 2016). However, earlier work examined cardiac *β*1‐adrenergic receptor densities in R6/2 and WT mice but did not see any genotypic differences (Mihm et al. 2007a). In HD patients, a similar decrease in HRV has also been reported during the presymptomatic and early stages of HD progression (Andrich et al. 2002; Kobal et al. 2004, 2010). Reduced HRV is generally considered an indication of poor cardiovascular health and a predictor for cardiovascular disease and mortality (Bigger et al. 1992; Buccelletti et al. 2009; Thayer et al. 2010).

The baroreceptor reflex is also a process dependent on autonomic function. Similar to the BACHD line (Schroeder et al. 2011), Q175 mice showed a dramatically blunted response in HR to the transient hypotension induced by NP suggesting that the sympathetic branch has impaired homeostatic reserve (Fig. 6). The primary deficit in the baroreceptor reflex in Q175 mice is unknown, as this process is mediated by various regions of the brain including the brainstem and hypothalamus, along with outputs from the ANS (Benarroch 1993; Ma et al. 2002; Kobal et al. 2004). In HD patients, the detection of alterations in the baroreceptor circuit, such as the vagal nuclei and cerebral cortex, have established a possible structural cause for this dysfunction (Benarroch 1993; Andrich et al. 2002; Ma et al. 2002; Bär et al. 2008; Kobal et al. 2010). Ideally, identification of the site of dysfunction could be used to devise an appropriate therapeutic approach to manage symptoms in HD patients. Prior studies reported HRV deficits during the Valsalva maneuver, hand‐grip test, and the head up tilt test in HD patients (Aminoff and Gross 1974; Den Heijer et al. 1988; Sharma et al. 1999; Bär et al. 2008). Furthermore, patients complain of dizziness and light‐headedness upon standing, suggesting they suffer from orthostatic hypotension due to autonomic dysregulation and a deficient baroreceptor reflex. (Kobal et al. 2004; Aziz et al. 2010). The sympathetic nervous system appears to be most impacted during the very early stages of HD (Kobal et al. 2004, 2010; Bär et al. 2008). As the disease advances, the parasympathetic activity progressively decreases as well (Sharma et al. 1999; Andrich et al. 2002; Bär et al. 2008).

As previously reported in the BACHD line as well as other HD models (Mihm et al. 2007a; Wood et al. 2012; Schroeder et al. 2016), the Q175 mice displayed an age‐dependent progression in heart dysfunction beginning at presymptomatic stages (3 months). Both the structure and function of the heart exhibited significant alterations due to both age and genotype (Fig. 7). The mutants’ hearts were smaller and less functional by 6 months of age (Table 3 & 5) and worsen with age. Histological analysis of the hearts also found that the mutant hearts showed localized fibrotic lesions that resembled those caused by myocardial infarction (Fig. 8C and D) and imply that prior obstruction of the blood supply to the heart caused local cell death. These fibrotic infarcted areas are the histological signature of heart attacks.

Cardiovascular complications are a leading cause of death in HD patients (Chiu and Alexander 1982; Lanska et al. 1988; Sørensen and Fenger 1992; Abildtrup and Shattock 2013). To date, several of the HD mouse models have been shown to exhibit cardiovascular dysfunction (Mihm et al. 2007a; Pattison et al. 2008; Kudo et al. 2011; Schroeder et al. 2011; Kiriazis et al. 2012; Wood et al. 2012; Buonincontri et al. 2014; Mielcarek et al. 2014). Mutant *Htt* is expressed in both the heart and brain (Strong et al. 1993), thus a critical question is whether the cardiovascular pathologies seen in HD are due to a deficit at the level of the cardiomyocytes or whether they are secondary to central nervous system dysfunction. For example, overexpression of an expanded polyQ track or *mHtt* in cardiomyocytes resulted in heart failure and a significantly reduced lifespan in both mice (Pattison et al. 2008) and flies (Melkani et al. 2013). So at least some of our findings may be due to cardiomyocyte‐specific deficits, e.g., the changes in the ECG waveform in young mutant mice. In addition, in HD patients, there is evidence of degeneration of brainstem nuclei, including those that control the regulation of heart function (Aminoff and Gross 1974; Izzo and Taylor 1999; Squitieri et al. 2001; Bär et al. 2008; Kobal et al. 2010; Rüb et al. 2014). So it is likely that there is an HD‐specific cardiomyopathology as well as cardiovascular pathology secondary to the HD‐driven damage to the ANS. Overall, this body of preclinical data suggests that monitoring of the cardiovascular system in HD patients should start at an early age so therapeutic interventions can by employed to slow the progression of these pathological processes and prevent early death. Most importantly, our data suggest that this early screening must include observations during the usual sleep hours of the day, as early anomalies may go undetected at the times of day that patients would usually interact with clinicians.

## Conflict of Interest

The authors have no competing interests.
